# Laypeople’s Online Health Information Search Strategies and Use for Health-Related Problems: Cross-sectional Survey

**DOI:** 10.2196/29609

**Published:** 2022-09-02

**Authors:** Yen-Lin Chiu, Chin-Chung Tsai, Jyh-Chong Liang

**Affiliations:** 1 Graduate Institute of Medical Education and Bioethics National Taiwan University College of Medicine Taipei Taiwan; 2 Program of Learning Sciences National Taiwan Normal University Taipei Taiwan; 3 Institute for Research Excellence in Learning Sciences National Taiwan Normal University Taipei Taiwan

**Keywords:** decision making, eHealth literacy, information search strategy, internet, patient, information-seeking behavior, laypeople, online health information, patient communication

## Abstract

**Background:**

With the increase in the use of the internet to search for health information about health-related problems, there is a need for health care professionals to better understand how their patients search for and use the online health information that may influence their medical decision making.

**Objective:**

The aims of this study are to explore laypeople’s online health information search strategies and examine the relationships between their search strategies and utilization behavior of online health information.

**Methods:**

Two scales, namely match and elaboration, were used to measure patients’ basic search strategies (ie, simple approach) and advanced search strategies (ie, integrative approach), respectively. In addition, the consultation scale was used to evaluate the participants’ use of online health information to consult doctors and others. A total of 253 outpatients without university education were purposely selected and surveyed. The participants were outpatients at a university-affiliated teaching hospital. Partial least squares-structural equation modeling (PLS-SEM) was performed to analyze the measurement model to specify the measurement validation. In addition, the structure model of PLS-SEM was evaluated to examine the path correlations between variables and to execute interaction effect and curvilinear relationship analyses.

**Results:**

The results of the path correlation analysis by PLS-SEM showed that both elaboration strategy (path coefficient=0.55, *P*<.001) and match strategy (path coefficient=0.36, *P*<.001) were positively correlated with consultation on online health information with doctors and others. In addition, interaction effect and curvilinear relationship analyses indicated that there was a significant interaction effect between elaboration and match on consultation (path coefficient=–0.34, *P*<.001) and a significant curvilinear relationship between match and consultation (path coefficient=–0.09, *P*=.046).

**Conclusions:**

Increasing patients’ exposure to online health information through both a simple search approach (ie, match strategy) and a complex search approach (ie, elaboration strategy) may lead them to appropriately use the information to consult doctors and others. However, the results of interaction effect and curvilinear relationship analyses highlighted the essential role of the elaboration strategy to properly locate, evaluate, and apply online health information. The findings of this study may help health care professionals better understand how to communicate with their patients through the health information on the internet.

## Introduction

### Background

With its convenient and widespread access to abundant information, the internet has become the major source for patients and the general population to retrieve health information [1]. As reported by the Pew Research Center, approximately 80% of American internet users search the internet for online health information [2]. In Taiwan, it is estimated that 83.4% of residents aged 12 years and above have internet experience [3]. As reported by the Taiwan National Health Interview Survey, 1766 (64.4%) of the 2741 surveyed individuals used the internet to search for online health information or services [4]. The issues regarding online health information–seeking behaviors of patients have attracted a great deal of attention, since the health information located on the internet obviously influences patients’ medical decision making [5-7].

Having better access to health information on the internet provides internet users with more possibilities to actively manage their own health and medical utilization behaviors [8]. The internet is regarded as a powerful and influential tool through which retrieving online health information may benefit patients’ empowerment, well-being health change, and healthier behaviors [9,10]. Compared to infrequent users, frequent internet users prefer more health-related information and decision making and the internet enables them to make more informed medical decisions [11]. In addition to medical decision making, online health information influences patients’ communication with physicians [12]. The result of a systematic review study showed that online health information improves patient-physician relationships as patients gain better access to online health information and discuss it with their physicians [13].

Despite the use of the internet to search for health information making internet users more knowledgeable, patients seldom discuss the information they find on the web with their doctors [14]. The credibility of the diverse range of health information on the internet has been of great concern, as its inappropriate use may be potentially harmful to patients’ health and waste medical resources [15,16]. In sum, online health information without verification by experts could generate misinformation and inappropriate health behaviors and hinder the physician-patient relationship [16,17].

According to systematic review studies, it has been concluded that the overall quality of online health information remains problematic and should be considered [18,19]. However, a high percentage (77%) of internet users tend to search for health information through search engines due to the decentralized nature of the internet [2]. An observational study on health information–seeking behaviors showed a high tendency of using search engines to look for health information [20]. As indicated by an experiment, the most popular method for seeking health information was to rely on the results of only 1 search engine page and to use unaccredited information to answer health questions without comparing and justifying them with other sources [5]. Because of the high heterogeneity of online health information sources, rather than merely relying on the first few results provided by the search engine, the need to check certain information against other information sources while searching for online information about medical problems has been strongly recommended [21,22]. The impacts of internet search strategies on information retrieval and how patients use online health information have been of great concern; however, the information search strategies for health-related problems have seldom been studied [7,23]. As suggested, there is a need to conduct more in-depth surveys to better understand how online health information–seeking behaviors influence the use of information in health-related decision making [24].

With respect to the role of education in the use of the internet for health information searching, it was reported that higher education is significantly connected to a higher probability of using the internet as the first source of health information [1]. The results of a population-based survey showed that respondents with lower education levels less frequently access health information from internet websites, while individuals with university or higher education more frequently search the internet for health information [10,25]. Regarding the use of online health information, the role of education has been linked to the use of credible information in health-related decision making [26]. While looking for health care providers to solve their medical problems, adult individuals with less formal education are less likely to use online resources to consult online rankings and reviews of doctors, hospitals, drugs, and medical treatments [27]. In a study on health information–related seeking and sharing behaviors among baby boomers and older adults, the results showed that college graduates and postgraduates are more likely than non-high-school graduates to seek and share health information over the internet [24].

According to a systematic review on studies that measured online health information usage, it was found that online health information can support desired health decisions, including increasing professional visits, asking questions during medical consultations, and adhering to physicians’ advice [28]. This review paper suggested that future studies strictly validate instruments for investigating online health information–seeking behaviors and carefully examine their impacts on health decisions. Using the concept analysis methodology, 1 study conducted a systematic review on the past 10 years of research to analyze the concept of health information–seeking behavior. The concept analysis results pointed out that the internet has become a common and preferred channel for retrieving health information. In addition to the importance of investigating how individuals from different communities seek information on the internet, the results of this study highlighted the lack of scales that can further measure and understand health information–seeking behaviors. They also concluded that there is a need to advance individuals’ ability to adequately acquire online health information and properly act on the acquired information to make health decisions [29].

### Research Purposes

Low levels of education have been correlated with undesirable online health information–seeking behaviors [24,27]. Research on the health information–seeking behaviors of the general population without a university education has been an issue of concern [22]. In addition, it was indicated that laypeople without a university background may receive less training in information search strategies and have difficulties searching for health information on the internet [5]. However, it was argued that either simple or complex search strategies would benefit general health information seekers to gather useful health information [5,20]. However, it was reported that those with low educational levels may not benefit from online health information, since they do not access alternative health information from health care providers [30]. As suggested, it is a major topic to explore how laypeople conduct either basic or advanced search strategies to obtain online health information to investigate and solve their health problems [20,31].

Therefore, this study examined in which manners patients without a university degree search for health information over the internet and how they use that information to answer their health-related questions. Thus, the correlations between patients’ health information search strategies and utilization behaviors were explored. Since the variables of search frequency, age, and sex were regarded as influential demographics in patients’ health information–seeking behaviors as well as doctor-patient consultations [32,33], these variables were also measured and recruited in the analyses and treated as control variables. Based on the aforementioned objectives, this study aimed to examine the following research questions:

- Question 1: Are there correlations between laypeople’s health information search strategies and their health information utilization?
- Question 2: Are there interaction relationships between health information search strategies and health information utilization?
- Question 3: Are there curvilinear relationships between health information search strategies and health information utilization?

## Methods

### Recruitment

To examine laypeople with a low-level education background, a probable sample of outpatients without university education was purposefully selected and surveyed in a large-scale, university-affiliated teaching hospital. The criterion for recruiting participants was having experience of searching for online health information. All the participants surveyed were patients who visited an outpatient clinic for health-related problems and consulted a doctor about their problems. All the participants voluntarily participated in this study by responding to the survey. Informed consent for the survey was obtained from individual participants. In addition, their privacy has been strictly protected.

### Instruments

#### Procedure for Developing and Validating the Measurements

According to the process suggested, the measurement development of this study was conducted in several steps involving theoretical and practical considerations [34]. With respect to the theoretical aspect, 2 measurements, the Information Commitment Survey (ICS) and Online Health Information Utilization (OHIU), were adopted from previous works that have involved clearly conceptual definitions and a theoretical basis for these measurements [7,35]. After receiving permission from the corresponding authors of these studies, the Chinese versions of the ICS and the OHIU were obtained and used in this study. Next, the wording of the items relating to the elaboration strategy, match strategy, and consultation were carefully modified to assess individuals’ opinions on searching for and using online health information. To ensure content validity, we requested 2 medical experts and 1 expert in information science to evaluate the correspondence between the individual item and its theoretical construct. In addition, we purposively recruited 10 representative participants in a pilot test to subjectively check whether the wording and readability of the ICS and OHIU were appropriate. Finally, we conducted partial least squares-structural equation modeling (PLS-SEM) to analyze the measurement model and examine the reliability, discriminant validity, and convergent validity of the measurements.

#### Demographic Variables

Demographic variables, including age, sex, and search frequency, were measured and recruited in the statistical analyses. Age was the participants’ actual age. For sex, males were coded as 1, while females were coded as 2. The search frequency, that is, the patients’ frequency of using the internet to search for health information for health-related problems, was measured with a 6-point scale ranging from 1 (rarely) to 6 (always).

#### Information Commitment Survey

Two constructs retrieved from the ICS signified web users’ information search strategies, namely the elaboration strategy and the match strategy [34,35]. These 2 constructs were modified and used to assess patients’ online information search strategies for answering their health-related questions. These measurements were evaluated with a 6-point Likert scale ranging from 1 (strongly disagree) to 6 (strongly agree), indicating participants’ opinions on each item of the search strategy. The details of the elaboration and match strategies are as follows.

- Elaboration as a search strategy (elaboration): evaluating the extent to which web users have metacognitive thinking and integrate information from diverse websites to find the best solution to fulfill their purposes. Example item: I can integrate the information retrieved from various websites.
- Match as a search strategy (match): assessing the extent to which web users wish to find a few websites containing fruitful and relevant information to match their searching purposes. Example item: I wish to find a single website containing the most fruitful information.

#### Online Health Information Utilization

The online health information consulting scale, named consultation, which is a subscale of the OHIU questionnaire, presented patients’ behaviors of using the health-related information retrieved from the web to consult doctors, experts, and relatives [7]. The items of consultation were measured with a 6-point Likert scale ranging from 1 (strongly disagree) to 6 (strongly agree), indicating participants’ opinions on their consulting behaviors. The definition of consultation is as follows:

- Consulting scale (consultation): measuring the extent to which patients consult others about the online health information they retrieve. Example item: I will discuss with a physician the issues regarding the medical information retrieved on the internet.

### Data Analysis

Statistical software packages for social science SPSS Statistics version 22 (IBM Corp) and SmartPLS3 (SmartPLS GmbH, Germany) were used to conduct statistical analyses. Using partial least squares-structural equation modeling (PLS-SEM) analysis, the measurement model of 2 instruments and the structural model of the research hypotheses were examined based on the 2-stage procedure recommended by Hair et al [36]. The statistical software SmartPLS3 was used to execute the PLS-SEM procedure. First, this study evaluated the reliability and validity of the ICS and OHIU instruments, including factor loadings, composite reliability (CR), average variance explained (AVE), and the Fornell-Lacker criterion [37]. Next, we executed path correlation analysis to examine the relationships among the participants’ age, sex, search frequency, elaboration, match, consultation, moderating term of elaboration and match, quadratic term of elaboration, and quadratic term of match. *P* values less than .05 indicated significant loadings and significant correlations between variables. Moreover, CR values greater than 0.7 and AVE values greater than the threshold value of 0.5 were considered as having adequate construct reliability and acceptable convergent validity, respectively [38].

### Ethical Considerations

This study was exempt from Institutional Review Board oversight in accordance with Article 5 of the Human Subjects Research Act of the Ministry of Health and Welfare, Republic of China (Taiwan) [39], and the “Scope of Human Research Cases Exempt from Ethical Review Board Review” announced by Ministry of Health and Welfare, Taiwan on 5 July 2012, pursuant to Wei-Shu-Yi-Zi (#1010265075) [40].

The research involved the use of questionnaires and survey procedures and was conducted in a public setting. The information obtained was recorded in such a manner that human subjects cannot be identified, directly or through identifiers linked to the subjects. Informed consent was obtained from all participants involved in the study, and the participants were subjected to no medical interactions or interventions other than ongoing usual care. The study was also conducted in accordance with the ethics standards required by the Declaration of Helsinki issued in 2013.

## Results

### Participants

A sample of 253 outpatients without a university academic degree was recruited for this study. The participants included 134 (53%) males and 119 (47%) females, who were outpatients at a university-affiliated teaching hospital in the northern area of Taiwan. Their average age was 45.73 (range 30-69) years.

### Results of Correlation Analysis

Table 1 provides the means and SDs of the variables and the Pearson correlation coefficients between them. As shown in Table 1, elaboration was linked to age (*r*=0.17, *P*<.01) and search frequency (*r*=0.24, *P*<.001) with positive correlation coefficients. In addition, both elaboration strategy (*r*=0.55, *P*<.001) and match strategy (*r*=0.31, *P*<.001) were positively correlated with consultation. That is, patients with high intent to conduct elaboration and match searches were more likely to consult others about the online health information they retrieved.

**Table 1:** Means (SDs) and correlations of variables.

Variables	Mean (SD)	Correlation			
		Age	Search frequency	Elaboration	Match
Age	45.73 (7.70)	N/A^a^	N/A	N/A	N/A
Search frequency	3.41 (1.07)	–0.07	N/A	N/A	N/A
Elaboration	4.68 (0.74)	0.17^b^	0.24^c^	N/A	N/A
Match	4.36 (0.84)	0.11	–0.06	0.09	N/A
Consultation	4.38 (1.07)	0.18^b^	0.09	0.55^c^	0.31^c^

^a^N/A: not applicable.

^b^*P*<.01.

^c^*P*<.001.

### PLS-SEM Analysis of the Measurement Model

PLS-SEM analysis of measurement model showed that the 9 items of 3 factors (elaboration, match and, consultation) had significant and satisfactory factor loadings ranging from 0.60 to 0.92. The CR value of each construct was fairly good, ranging from 0.81 to 0.89. Moreover, the AVE values were larger than the threshold value of 0.5, ranging from 0.59 to 0.74, showing acceptable convergent validity [41]. Based on the Fornell-Lacker criterion, the square root of the AVE for each factor (ranging from 0.77 to 0.86) was higher than the corresponding interfactor correlations (ranging from 0.09 to 0.55), suggesting reasonable discriminant validity [37]. For details of the measurement model analysis, please refer to Multimedia Appendix 1.

### Path Correlation Analysis of the Structural Model

Combined with main variables, demographic variables, moderating term, and 2 quadradic terms, path correlation analysis was performed using SmartPLS3. The main variables containing elaboration, match, consultation, and demographic variables, including sex, age, and search frequency, were involved in the structural model to evaluate the path coefficients between the variables. To further examine the nonlinear effects of elaboration and match on consultation, following the procedure suggested, we used the 2-stage approach to create a moderating term (interaction effect between elaboration and match) and 2 quadratic terms (quadratic effects of elaboration and match) on the basis of standardized data [42].

**Figure 1:**
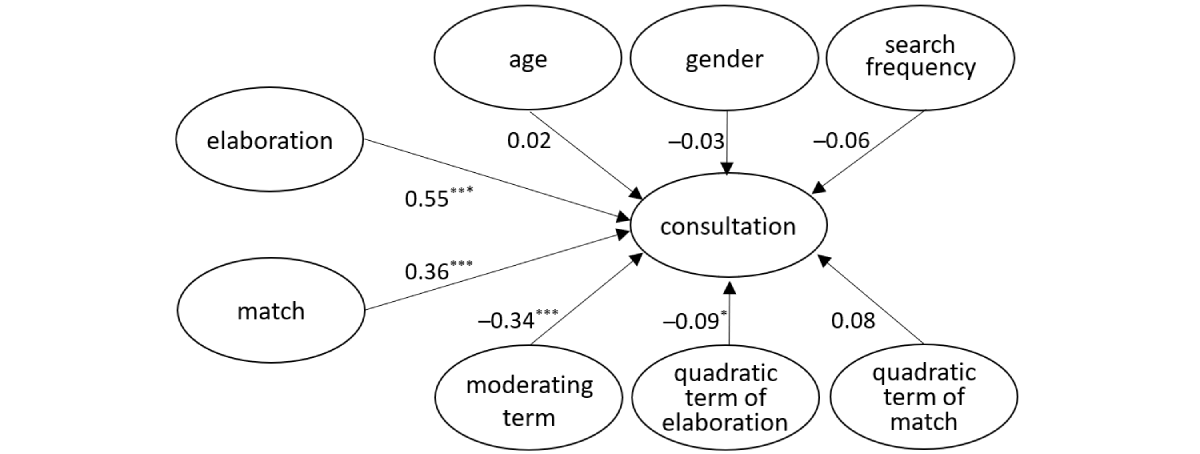
Path correlations of the structure model. *P<.05, ***P<.001

Figure 1 presents the path coefficients of the structural model. The elaboration (path coefficient=0.55, *P*<.001) and match (path coefficient=0.36, *P*<.001) showed positive correlations between consultation, while the moderating term (path coefficient=–0.34, *P*<.001) and quadratic terms of match (path coefficient=–0.09, *P*=.05) showed negative correlations with consultation. Regarding demographics, sex, age, and search frequency did not have significant correlation with consultation. Overall, the R^2^ value for consultation was 0.49, while the adjusted R^2^ value was 0.47. In addition, the f^2^ values of elaboration, match, moderating term, and quadratic terms ranging from 0.18 to 0.43 were higher than 0.025, showing large effects of independent variables [42]. Moreover, the values of the variance inflation factor (VIF) for independent variables ranged from 1 to 2.84, indicating that there was no problem of collinearity [41].

**Figure 2:**
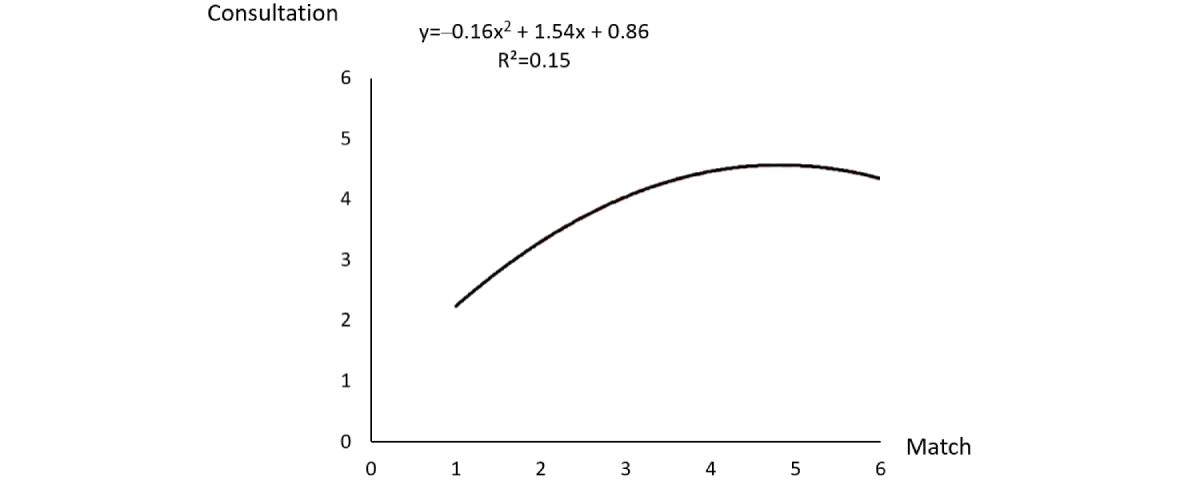
Nonlinear graph of the match variable.

To further illustrate the curvilinear relationship of match with consultation, we used the means of latent variables calculated by PLS to estimate the quadratic equation of consultation on match. The scatter plot with its trend curve is plotted in Figure 2. As presented, the coefficient of x was positive, while the coefficient of X^2^ was negative, indicating a concave downward relationship between match and consultation. That is, an increase in match had an initial positive effect on consultation, but the effect became weaker and even changed direction when match reached a high level, suggesting that match has a decrement of positive effect on consultation.

**Figure 3:**
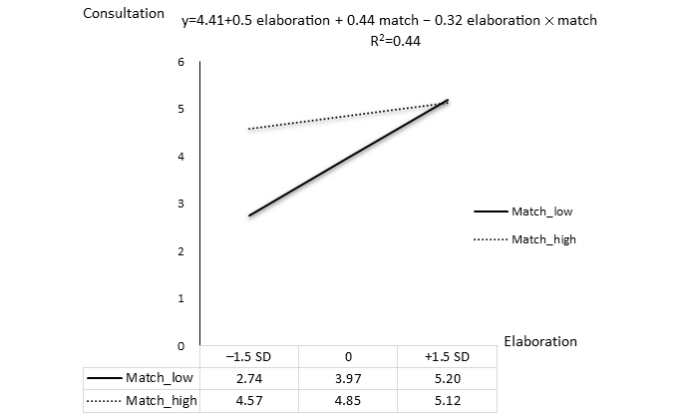
Interaction effect between match and elaboration on consultation.

To better understand the interaction effect between elaboration and match, we used the standardized latent means of elaboration and match calculated by PLS to analyze the regression of consultation for representative groups. As suggested, the low- and the high-match group were chosen at low (–1 SD from the mean) and high (1 SD from the mean) values of match, respectively [43,44]. To observe the crossover interaction, the consultation scores for the low- and high-match groups were calculated at a low level (–1.5 SD) and a high level (1.5 SD) of elaboration, respectively [44]. Next, the predicted values for each group were produced by multiplying the respective unstandardized regression coefficients for each variable at an appropriate value (eg, high match=1, high elaboration=1.5). Figure 3 shows the plot of interaction between match and elaboration. The solid line is for the low-match group (at a value of –1), while the dotted line is for the high-match group (at a value of 1). The result indicates that elaboration had a positive effect on consultation for both the low- and the high-match group. However, the slopes show that when the match was low, the effect of elaboration on consultation was stronger than that of a high match. Furthermore, the crossover interaction shows that when elaboration was low, the high-match group had a higher consultation score than that of the low-match group. On the contrary, when elaboration was high, the low-match group had a higher consultation extent than that of the high-match group.

## Discussion

### Principal Findings

#### Role of Education in Health Information Seeking

It has been reported that online health information may potentially benefit individuals by making them better informed, resulting in more effective health outcomes; on the contrary, misinformed health information may result in inappropriate use of medical resources [15]. In addition, studies have indicated that individuals with lower education levels are less likely to access websites for health information and show unsuitable utilization behaviors, while people with university degrees more frequently access online health information using complex and expanded information search strategies [10,25,26]. Therefore, the population without university degrees has been regarded as an important target group to examine their online health information navigation behaviors [22]. Accordingly, the results of this study may provide expanded views on the online health information search behaviors of those with low educational backgrounds.

#### Positive Influences of Health Information Searching on Consultations

As can be seen from the results presented in Table 1, the correlations between information search strategies and consultation showed that both match strategy and elaboration strategy have positive influences on the usage of online health information to consult others. That is, no matter what search strategy the patients used to gather online health information, they were willing to further discuss the information with medical experts or others. Despite an advanced search strategy, such as an analytic approach, being considered an important factor connected with accurate search results, it was emphasized that simple strategies, such as the browsing approach, which may be efficient and successful, need not necessarily be rejected [23]. As was expected, patients with more exposure to health information through information communication technology (both advanced and simple approaches) were more likely to perform healthier behaviors, suggesting a potential way for health care professionals to encourage their patients to access online health information and communicate health information with them through digital media [10].

#### Curvilinear Relationship of Match With Consultation

Curvilinear analysis of the match strategy indicated that it was positively linked to consultation willingness, but the correlation became weaker and even changed direction as the match strategy reached a high level. That is, accessing online health information through the match strategy is necessary and helpful for consulting health care professionals about the retrieved information, but too much use of this simple approach may disadvantage consultation behaviors. Similarly, it was reported that health information seekers without medical expertise are more likely to use search engines to perform a simple search; although it would be useful to engage them in the information discovery process, it also may become a barrier to further obtaining the most suitable solution [20].

Based on the theory of planned behavior (TPB), it was indicated that abundant information may overload information seekers and result in their psychological ill-being (eg, depression and anxiety), which may discontinue their intention to use the online health information [45]. Accordingly, it may explain why the match strategy has a positive influence on consultation behavior, implying that gathering relevant information from a few resources may support information seekers’ continuous use of online health information. Nevertheless, an overwhelming amount of information retrieved by the match strategy without the skills of evaluating and integrating such information may discourage its continuous use. To summarize prior research, there are interesting findings on health information seekers’ health information–seeking behaviors and responses to the gathered information [5,20,31]. Simple lookup search strategies may have both advantages and disadvantages for individuals’ health information–seeking behaviors [5,20,31]. Furthermore, it was demonstrated that multiple health information sources through an instant search approach can lead to information overload and result in information avoidance, suggesting the need for training on advanced health information–seeking skills to manage and integrate diverse information sources [46].

#### The Elaboration Strategy Is Essential to Desired Health-Seeking Behavior

As laypeople do not have medical expertise, they tend to adopt basic search strategies to look up online health information for retrieving facts and answering health questions [20,47]. However, the correlation analyses in this study showed that the elaboration strategy has more positive influences on consultation than the match strategy. In addition, interaction effect analysis indicated the important role of the elaboration strategy in reinforcing patients’ willingness to further consult medical experts or others with the online health information they have found, especially patients with a tendency to adopt a low-match strategy. In conclusion, the elaboration strategy may be a better choice than the match strategy through which to encourage patients to gather and integrate numerous types of health information and use such information appropriately. To further understand and interpret health information, health information seekers have to adopt advanced search strategies to scan and justify the search results [47]. As suggested, patients and their relatives were encouraged to conduct more advanced search strategies to recognize credible and appropriate health information sources [32].

#### The Importance of eHealth Literacy for Advancing Health-Seeking Behavior

In Taiwan, an investigation on health information–seeking behaviors showed that internet users with high educational levels (university and above) are more likely to use the internet for health information searching. Regarding the effects of health information searching, a majority of the respondents used such health information to ask physicians questions and to make decisions on disease treatment and whether to consult a physician [4]. Therefore, online health information seeking can be regarded as a channel through which health care professionals can enhance patient-physician relationships and help patients by recommending credible health information sources.

In conclusion, there is a need to investigate how to stimulate internet users with low educational levels (without a university education) to use health information to consult health care professionals and to have positive effects on their treatment decisions and health outcomes. Based on the results of this study, health care professionals may better know how medicine-related information search strategies (ie, match and elaboration strategies) can benefit patients with low educational levels when turning to the internet for making health decisions [26]. In sum, this subpopulation (those with less education) may benefit from online information only when they have access to alternative health information sources, such as health care providers [30].

When compared with the low-level-eHealth-literate group, high-level-eHealth literate individuals who have a good ability to seek, locate, evaluate, and apply online health information were recognized as more frequent health information seekers and were better at using effective online health information search strategies to address their health concerns [5]. As suggested, improving eHealth literacy may promote individuals’ use of effective online information-seeking strategies and identify high-quality health information sources. In the case of this study, for patients in both the low- and the high-match group (in particular, those with a low tendency to adopt the match strategy), developing their eHealth literacy may encourage their intent to use the elaboration strategy and consult health care professionals.

### Limitations

Several limitations of this study should be noted. First, this study targeted laypeople without a university education in order to examine their online health information search behaviors rather than other populations with a university degree or higher educational background. That is, the results of this study should be cautiously interpreted and inferences should be made with care. The second limitation is the sampling method used in this study. The participants included in this study were purposefully recruited from 1 university-affiliated teaching hospital rather than from other clinical settings, such as small hospitals or private clinics. Therefore, the generalizability of the study results is limited to other clinic settings and regions in Taiwan. Third, instead of objective data, such as log files, the data of this study were collected from patients’ subjective opinions and attitudes. Thus, the self-reported bias should be considered. Finally, a few predicting factors, including age, sex, search frequency, and search strategies, were explored in this study and recruited in the regression analysis model. Although the results of PLS path analysis indicated that a high proportion of variances was explained by the predictors, there is still a need to further consider other predictors or confounding factors, such as severity of illness and accessibility of medical resources, which may influence how patients use online health information.

### Conclusion

Although there are challenges for laypeople, who are not medical experts, and who do not have a university degree to properly access and evaluate the credibility and accuracy of health information retrieved from the internet [10,22], understanding their online health information search strategies and use of such information may help health care professionals better know how to lead their patients to appropriately search for and communicate about online health information with medical experts. Certainly, the internet is an essential tool through which patients may approach the low-cost wealth of health information; however, it is an additional source of health information, which should not necessarily replace traditional health information offered by health care professionals [9].

Based on the findings of this study, we provided practical suggestions in several aspects. As suggested, the public population and patients were encouraged to gather health information from multiple sources, including medical experts’ advice, as well as alternative opinions from the internet [7,25]. It has been indicated that patients use online medical information to integrate with advice from friends, family, and physicians in order to confidently make their medical decisions [12]. According to the results of this study, patients without a university degree should be supported to obtain more exposure to online health information through both complex and simple search approaches, which in turn may induce them to consult medical experts about such information. In addition, it was suggested that health care providers should recognize that their patients are using the internet as a medical information source, and should be prepared to help patients to carefully identify the quality of online health information and appropriately use such information [48]. That is, medical professionals must be aware that they are eligible to direct patients’ health information search behaviors and empower them to engage in an informed and active way in their own medical decision-making process. Finally, we recommend that health care providers offer high-quality information on well-designed medical websites. To assist patients in adopting simple searches and becoming advanced explorers, there is a need to provide better information tools and quality content for them to surf the internet full of rich information and many pitfalls [47]. While conducting a heuristic search, patients sometimes reject credible websites with high-quality content due to poor visual appeal and unclear interface design [12]. In other words, well-designed websites built by medical professionals containing a clear interface and quality health information can draw the attention of patients and lead them to access trustworthy information while looking up health information on the internet.

Meanwhile, the results of the interaction and curvilinear analyses suggested that the elaboration strategy is a more recommendable approach than the match strategy through which patients are more likely to use online health information to consult with their doctors or others about their health-related problems. To stimulate patients’ online health information search strategies in more advanced ways, it has been suggested that advancing patients’ eHealth literacy (ie, ability to search, locate, evaluate, integrate, and apply electronic health information) may support them to conduct appropriate information search strategies, justify reliable and useful information, and use such information in an effective manner [5,45].

In summary, this study acknowledges how patients without a university degree search for health information over the internet, how they share the information with doctors and others, and how to guide them to accurately use the information sources. As patients have better access to additional medical advice over the internet and can discuss such information with health care professionals, they are expected to be more involved in appropriate health information and engaged in their medical decision making.

## Appendix

Multimedia Appendix 1 Details about the questionnaire and its validation.

Multimedia Appendix 2 Cover letter describing informed consent.

## Abbreviations

AVE: average variance extracted
CR: composite reliability
ICS: Information Commitment Survey
OHIU: Online Health Information Utilization
PLS-SEM: partial least squares-structural equation modeling
