# Supplementary material for: Laypeople’s Online Health Information Search Strategies and Use for Health-Related Problems: Cross-sectional Survey
Source: J Med Internet Res. 2022 Sep 2;24(9):e29609. doi: 10.2196/29609 (PMC9482072; doi:10.2196/29609)
Supplement: Multimedia Appendix 1 [file jmir_v24i9e29609_app1.docx]

## Results of PLS-SEM measurement model analysis.

| Items | Loadings | Composite Reliability (CR) | Average Variance Extracted (AVE) | Rho value | α value |
| --- | --- | --- | --- | --- | --- |
| EL1 | 0.85 | 0.88 | 0.70 | 0.79 | 0.79 |
| EL2 | 0.88 |  |  |  |  |
| EL3 | 0.79 |  |  |  |  |
| MA2 | 0.80 | 0.81 | 0.59 | 0.79 | 0.71 |
| MA1 | 0.89 |  |  |  |  |
| MA3 | 0.60 |  |  |  |  |
| CO1 | 0.89 | 0.89 | 0.74 | 0.85 | 0.82 |
| CO2 | 0.76 |  |  |  |  |
| CO3 | 0.92 |  |  |  |  |

EL1: I am used to summarizing my own information with numerous types of medical information retrieved from the Internet.

EL2: While searching for medical information, I will integrate information from various websites or webpages.

EL3: I will compare diverse medical information which is located on different websites.

MA1: Usually, I merely use one search engine to find the medicine-related webpages which best meet my need.

MA2: I would judge whether to read the content of a website based on the degree of relevance provided by a search engine.

MA3: I want to find the one web page which contains the most abundant medical information.

CO1: After discussing the online medical information with experts I will make my medical decisions referring to the discussion.

CO2: I will discuss with a physician the issues regarding the medical information retrieved on the Internet.

CO3: I will discuss relevant issues of medical information retrieved on the Internet with my family or friends.

## Results of the discriminant validity analysis.

| Factors | Consultation | Elaboration | Match |
| --- | --- | --- | --- |
| Consultation | **0.86** | — | — |
| Elaboration | 0.55 | **0.84** | — |
| Match | 0.31 | 0.09 | **0.77** |

Note: The square root values for average variance explained estimates (in boldface) are presented on the diagonal, while the correlations between factors are below the diagonal. — Not applicable.
