# Supplementary material for: Laypeople’s Online Health Information Search Strategies and Use for Health-Related Problems: Cross-sectional Survey
Source: J Med Internet Res. 2022 Sep 2;24(9):e29609. doi: 10.2196/29609 (PMC9482072; doi:10.2196/29609)
Supplement: Multimedia Appendix 2 [file jmir_v24i9e29609_app2.docx]

Informed consent

Dear Patient:

This is a research project being conducted by Dr. Chiu Yen-Lin and her colleagues. The purpose of this questionnaire is to explore your experience about the use of the internet to search for health information about health-related problems. The results of this survey will help health care professionals to better understand how their patients search for and use the online health information that may influence their medical decision making.

Your participation is voluntary. You may choose not to participate in this study. If you decide to participate in this research, you can interrupt and withdraw from the survey at any time. Your declination and withdraw will not injure your personal rights.

If you agree to participate in this survey and complete the questionnaire, the information you fill in will be properly protected to ensure your personal privacy. Your responses will be confidential and we do not collect identifying information. We will do our best to keep your information confidential. The results of this study will be used for scholarly purposes only.

If you have any question about the research study, please contact Dr. Chiu YL (yenlin.address@gmail.com) to let us help with your queries. Thank you for your participation.

Best regards,

Chiu, Yen-Lin

Director of research project
